# Interobserver and Intraobserver Reliability of a Novel Classification System for Distal Radioulnar Joint Instability

**DOI:** 10.3390/life16020243

**Published:** 2026-02-02

**Authors:** Awad Dmour, Alexandra Maria Burlui, Bianca-Ana Dmour, Ștefan-Dragoș Tîrnovanu, Dragoș-Cristian Popescu, Mihaela Camelia Tîrnovanu, Liliana Savin, Mihaela Pertea, Tudor Cozma, Adrian Claudiu Carp, Paul-Dan Sîrbu, Bogdan Puha

**Affiliations:** Faculty of Medicine, Grigore T. Popa University of Medicine and Pharmacy, 700115 Iasi, Romania; dmour-awad@umfiasi.ro (A.D.); maria-alexandra.burlui@umfiasi.ro (A.M.B.); dragos.popescu@umfiasi.ro (D.-C.P.); mihaela.tirnovanu@umfiasi.ro (M.C.T.); liliana.savin@umfiasi.ro (L.S.); mihaela.pertea@umfiasi.ro (M.P.); tudor.cozma@umfiasi.ro (T.C.); adrian-claudiu.carp@umfiasi.ro (A.C.C.); paul.sirbu@umfiasi.ro (P.-D.S.); bogdan.puha@umfiasi.ro (B.P.)

**Keywords:** distal radioulnar joint instability, classification system, interobserver reliability, intraobserver reliability, distal radius fractures, Galeazzi fracture, Essex Lopresti injury

## Abstract

A clinically useful classification system requires precise definitions, reproducibility, and applicability across different levels of clinical experience. Distal radioulnar joint instability remains insufficiently represented in fracture based classifications, contributing to diagnostic uncertainty and variability in treatment strategies. This retrospective observational study assessed the interobserver and intraobserver reliability of a previously proposed classification system for distal radioulnar joint instability. Five orthopedic surgeons, including three board-certified specialists and two final year residents, independently evaluated forty five clinical cases comprising distal radius fractures, Galeazzi fractures, and Essex Lopresti injuries, using predefined clinical and radiological criteria. Interobserver agreement was analyzed using Fleiss Kappa statistics, while intraobserver reliability was evaluated after a two week interval using Cohen Kappa. Overall interobserver agreement was excellent, with a global Fleiss Kappa value of 0.87. Agreement was highest in Galeazzi fractures and lowest in Essex Lopresti injuries, reflecting increased diagnostic complexity in this subgroup. Specialists demonstrated higher agreement than residents. Intraobserver reproducibility was excellent, with identical classifications in 96 percent of reassessed cases. These findings indicate that the proposed classification system shows high reliability and reproducibility, supporting its potential clinical utility for standardized assessment of distal radioulnar joint instability and for guiding future clinical and biomechanical research.

## 1. Introduction

For a classification system to be clinically meaningful, it must be clearly defined, reproducible, and consistently applicable. When interobserver reliability is low, the interpretation of a classification becomes subjective, which can influence both diagnostic accuracy and therapeutic decision making [1,2]. Assessing agreement between observers therefore represents an essential step in validating any system intended for routine clinical use.

The distal radioulnar joint instability classification evaluated in this study was previously described in our earlier publication, in which its anatomical framework, clinical criteria, and grading rationale were presented in detail [3]. The present study represents a distinct methodological stage, focusing exclusively on the assessment of the reproducibility of this system. The classification stratifies distal radioulnar joint instability into three grades: grade 1, characterized by preserved joint alignment; grade 2, defined by subtle loss of joint congruency without gross instability; and grade 3, representing complete instability with loss of joint congruency.

The aim of this study was to determine the consistency with which the classification is applied by clinicians with different levels of experience, through an analysis of inter and intraobserver agreement [1,4]. Our objective was to identify the degree to which the proposed criteria are interpreted uniformly and to highlight any areas where ambiguity or variability may occur.

To quantify reproducibility, we used Fleiss Kappa, a widely accepted statistical measure for evaluating agreement in orthopedic classification systems involving multiple observers [5,6]. In addition, stability of ratings over time was assessed through a second evaluation performed after two weeks, without access to prior results, enabling calculation of intraobserver agreement [7,8]. These analyses allowed us to determine the reliability of the classification and to identify elements that may require clarification to ensure consistent application in clinical practice.

## 2. Materials and Methods

### 2.1. Study Design and Observers

This retrospective observational study aimed to evaluate the inter and intraobserver reliability of the new DRUJ instability classification. Five orthopedic surgeons participated as evaluators, consisting of three board certified specialists and two final year residents. All observers independently assessed the selected clinical cases using the proposed classification system. They had no access to the original diagnosis or treatment data and based their assessments exclusively on the available imaging together with the predefined clinical criteria.

For the analysis of interobserver reliability, the classifications assigned by each evaluator were compared to determine the degree of concordance. To examine temporal stability, each observer repeated the assessment after a two-week interval without access to their prior evaluations. This reassessment allowed the analysis of intraobserver reliability and provided information regarding the reproducibility of the system over time and the potential presence of individual variation in interpretation.

Before initiating the evaluation, all observers participated in a standardized training session designed to ensure uniform understanding and application of the classification. The session lasted 15 min, during this session, the evaluators reviewed the detailed definition of each instability grade, examined representative cases derived from the original classification description, and discussed the radiological and clinical criteria. The purpose of this preparatory stage was to reduce subjective variability and enhance the consistency with which the classification was applied.

This study was designed exclusively to assess interobserver and intraobserver reliability of the proposed distal radioulnar joint instability classification. No inferential analyses were planned or performed to evaluate clinical outcomes, prognostic performance, or treatment effectiveness.

### 2.2. Case Selection

**Figure 1:**
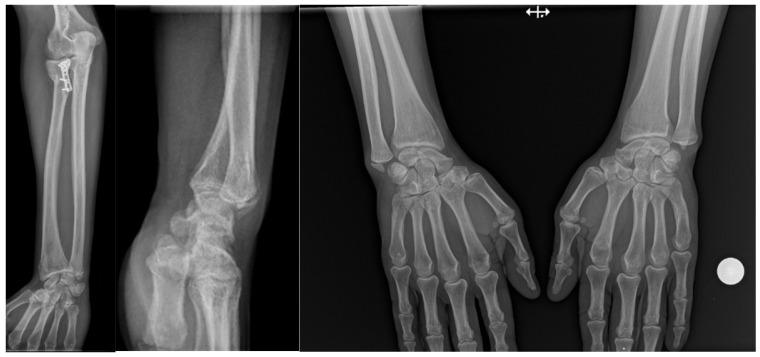
Essex Lopresti lesion treated with radial head plate fixation, associated with ulnar styloid fracture and dorsal displacement of the ulnar head. The increased DRUJ gap and positive ulnar variance compared with the contralateral side indicate a grade 2 DRUJ injury.

The study included forty-five patients diagnosed with injuries of the distal radioulnar joint and treated at Saint Spiridon Hospital in Iași, Romania. The cases were selected to represent the full spectrum of traumatic patterns associated with DRUJ instability [9,10]. Twenty patients had distal radius fractures, twenty presented Galeazzi fractures, and five were diagnosed with Essex Lopresti injuries (Figure 1).

Inclusion criteria were distal radius fractures, Galeazzi fractures, or Essex Lopresti injuries with available standard radiographs allowing assessment of distal radioulnar joint alignment and instability. Exclusion criteria were incomplete imaging, preexisting distal radioulnar joint pathology, prior wrist surgery, or bilateral injuries. Each patient contributed a single injured wrist to the analysis, and no case was included more than once.

**Figure 2:**
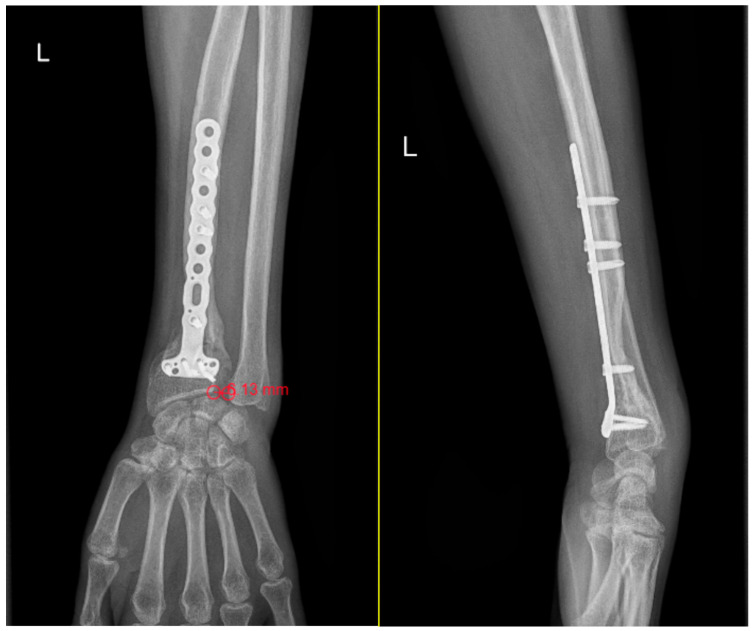
Measurements of DRUJ gap and ulnar variance on standard anteroposterior wrist radiographs. DRUJ distance greater than 5 mm (6.13 mm) following internal fixation of a distal radius fracture with a locked titanium plate. The increased gap is consistent with a grade 3 DRUJ injury, lateral view that shows neutral ulnar head orientation.

Evaluation criteria DRUJ injuries were classified using a combination of radiological and clinical parameters, with the objective of characterizing the severity of joint instability. From an imaging perspective, three key elements were assessed. The first was the DRUJ distance, with values greater than 5 mm considered suggestive of significant instability (Figure 2) [11,12]. The second was ulnar variance, which was regarded as normal when within ±2 mm [13,14,15]. The third was the orientation of the ulnar head on the lateral radiograph, which was categorized as neutral, dorsally inclined when the projection exceeded 6 mm relative to the radius, or volar inclination [16,17].

Clinical assessment of DRUJ stability included functional tests and objective parameters. The Press test was used to detect pain under loading conditions, indicating potential compromise of stabilizing structures [18,19]. The fovea sign assisted in differentiating patients with or without a triangular fibrocartilage complex lesion and provided important information regarding ligamentous integrity [11,20]. The ballottement test was used to determine whether the joint demonstrated normal stability or pathological translation consistent with instability [21].

**Figure 3:**
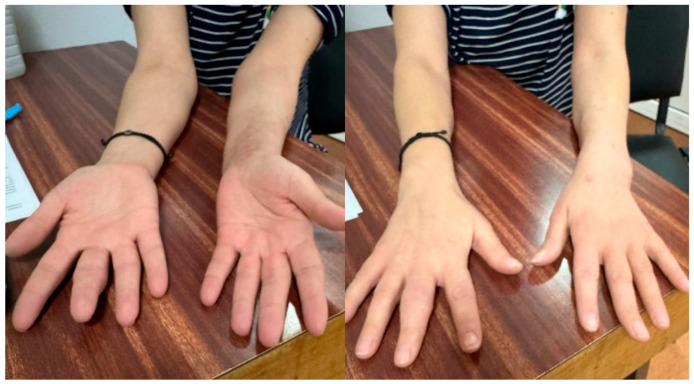
Limited supination in a patient with an initial grade 3 DRUJ injury, and normal pronation clinical aspect at 3 months postoperative.

Additional functional measures were recorded to provide a more complete understanding of wrist and forearm performance. Grip strength was compared with the contralateral limb as an objective indicator of functional impairment [22]. Pronation and supination ranges were documented to assess the impact of instability on forearm mobility (Figure 3) [20,23,24]. Pain intensity was quantified using the Visual Analog Scale, with patients rating their symptoms from 0 to 10 [25,26]. These complementary parameters contributed to a broader clinical context for interpreting the severity of DRUJ dysfunction.

### 2.3. Statistical Analysis

Statistical analysis was performed using IBM SPSS Statistics version 27. Fleiss kappa was used to evaluate global interobserver agreement among all five observers, whereas Cohen kappa was applied for subgroup analyses involving pairwise or two observer comparisons, in accordance with standard methodological practice for categorical reliability studies. Confidence intervals for kappa statistics were calculated using standard asymptotic methods implemented in SPSS, and rounded to two decimal places. According to standard interpretation thresholds, values above 0.80 represent excellent agreement, values between 0.60 and 0.79 substantial agreement, and values below 0.60 moderate or poor reliability [7,27,28].

To examine the influence of clinical experience on application of the classification, analyses were conducted both for the full group and separately for specialists and residents. Cases showing the greatest variability in assigned grades were identified in order to highlight potential sources of discrepancy. For intraobserver reliability, each evaluator reassessed the same cases after two weeks without access to their previous ratings. This second evaluation allowed measurement of the temporal consistency of the classification and provided insights into its stability when applied repeatedly by the same clinician.

## 3. Results

### 3.1. Global Interobserver Agreement

**Figure 4:**
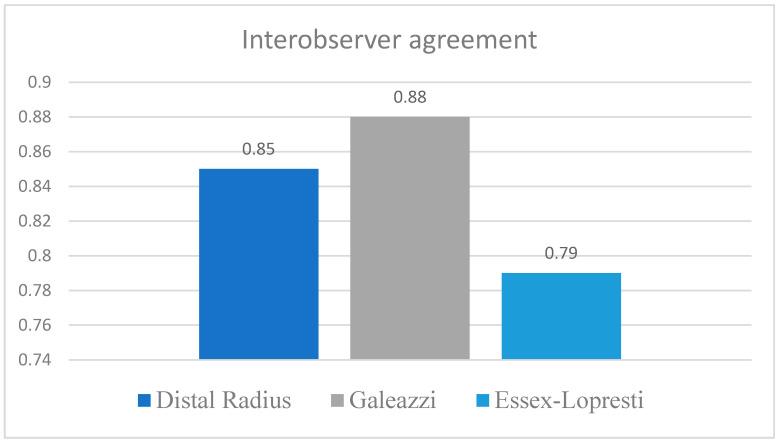
Interobserver agreement according to the type of DRUJ injury.

Interobserver agreement calculated using Fleiss Kappa demonstrated excellent overall concordance among the five evaluators, with a global kappa of 0.87 (SE 0.05, 95 percent CI 0.76 to 0.97). When analyzed by injury type, Galeazzi fractures showed the highest level of agreement (κ = 0.88), followed by distal radius fractures (κ = 0.85). The lowest reliability was observed in Essex Lopresti lesions, where Fleiss Kappa reached 0.79, reflecting the greater diagnostic complexity of this subgroup and the presence of subtle radiological and clinical findings that challenged consistent interpretation (Figure 4).

**Figure 5:**
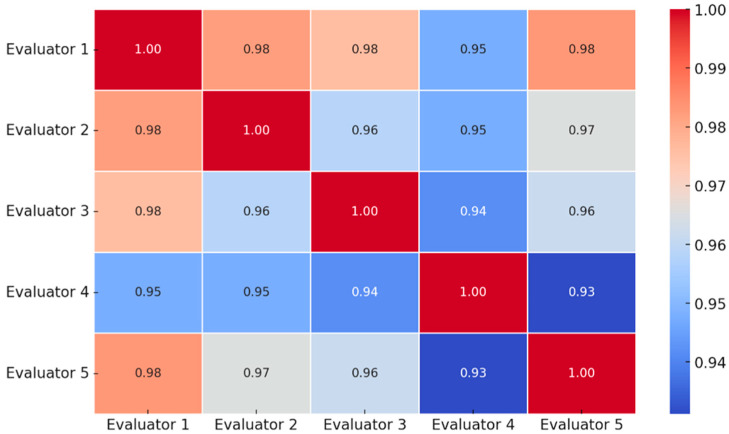
Pairwise interobserver agreement values between evaluators, visually highlighting concordance patterns identified in the kappa analysis.

Comparison of evaluator experience revealed that specialists achieved superior agreement relative to residents. Interobserver reliability among specialists was excellent, with a Fleiss Kappa of 0.92 (95 percent CI 0.84 to 1.00). Agreement between residents was substantial but lower, with a Cohen Kappa of 0.76 (95 percent CI 0.61 to 0.91) (Figure 5).

### 3.2. Cases with the Greatest Disagreement

**Figure 6:**
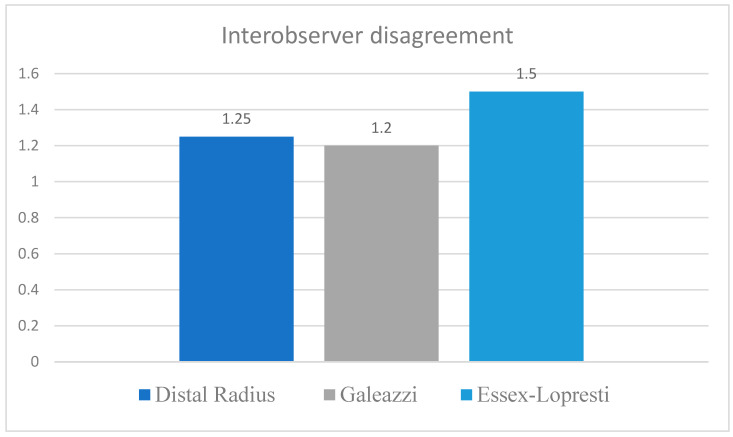
Interobserver disagreement according to the type of DRUJ injury.

The highest degree of disagreement occurred in Essex Lopresti injuries, followed by borderline distal radius fractures and less severe Galeazzi fractures. These findings were consistent with the corresponding Fleiss Kappa values recorded for each subgroup (Figure 6). Discrepancies emerged mainly in cases of marginal instability, particularly those in which the DRUJ gap measured between 1.5 and 2.7 mm and clinical signs were minimal or inconsistent. These measurements fall below the 5 mm threshold for marked instability and therefore represent borderline presentations that challenged discrimination between lower grades. Interpretation proved especially difficult in patients with slight ulnar inclination or borderline radiological misalignment, where observers differed in estimating the severity of instability. These cases highlight the importance of refining thresholds separating low grade from moderate instability to improve consistency.

Most cases with high variability were observed among Essex Lopresti injuries and within the mild end of the Galeazzi fracture spectrum, both of which are associated with subtle or progressive patterns of distal radioulnar joint dysfunction that may account for the observed heterogeneity in interpretation.

### 3.3. Correlation Between DRUJ Classification and Clinical Parameters

Although the primary objective of the study was to assess classification reliability, several clinical parameters were recorded to provide descriptive context for the observed instability grades. Larger DRUJ distances were more frequently observed in higher instability grades, consistent with the imaging thresholds defined in the classification. Patients classified as grade 3 demonstrated lower grip strength, more pronounced limitations in pronation and supination, and higher pain intensity on the Visual Analog Scale. These associations are reported descriptively to illustrate internal coherence between radiological severity and clinical presentation. They were not subjected to inferential analysis and were not used to validate outcomes or prognostic performance, as such analyses fell outside the scope of the present study.

### 3.4. Intraobserver Agreement

Intraobserver reproducibility was excellent. After a two-week interval, repeated evaluations remained identical in 96 percent of cases. Discrepancies occurred only in two borderline cases in which ratings alternated between grade 2 and grade 3. These differences are consistent with expected clinical variability when evaluating marginal instability features.

Intraobserver reliability remained high across all evaluators, with Cohen Kappa values ranging from 0.84 to 0.94. Specialists demonstrated greater temporal consistency, with kappa values between 0.90 and 0.94, whereas residents showed slightly lower but still excellent reproducibility, with values between 0.84 and 0.87. All confidence intervals remained above 0.73, confirming robust repeatability of the classification and supporting its stability when applied by the same observer under comparable conditions. The limited variability observed was confined to borderline cases and reflects expected interpretative uncertainty when radiological and clinical signs fall near threshold values rather than inconsistency of the classification criteria.

## 4. Discussion

### 4.1. Interpretation of the Results

The proposed classification for distal radioulnar joint instability demonstrated a high level of interobserver reliability, supporting its potential utility for standardized clinical assessment. The study included both board certified orthopedic surgeons and senior residents, ensuring a distribution of experience comparable to that used in other validation studies of orthopedic classification systems [1,4,29]. Agreement among specialists was almost perfect (κ = 0.92), while residents achieved substantial agreement (κ = 0.76). These findings indicate that the classification can be applied consistently even by clinicians with less extensive experience.

The difference observed between the two groups suggests that a structured training process may further enhance consistency, particularly in the assessment of borderline lesions. Evaluator 4, one of the residents, contributed the greatest variability, especially in cases with a DRUJ gap between 1.5 and 2.7 mm. This pattern highlights the need to refine the current thresholds separating mild from moderate instability [30]. The difficulty encountered by less experienced evaluators in interpreting subtle radiological findings is consistent with the observations reported by [31]. Their study comparing diagnostic accuracy between radiology specialists and residents demonstrated that specialists more frequently identified subtle abnormalities (*p* = 0.04). These data underscore the importance of clinical experience in detecting discrete pathological changes in anatomically complex regions. In the context of the present DRUJ classification, the findings support the need for targeted instruction focused on borderline cases where radiological and clinical signs are minimally expressed [31].

The greatest discrepancies were identified in Essex Lopresti injuries, where the Fleiss Kappa value of 0.79 was the lowest among subgroups. This result is influenced by the limited number of cases (n = 5) and by the higher diagnostic complexity intrinsic to this injury pattern [32,33]. Cases with marginal DRUJ instability, defined by gaps between 1.5 and 2.7 mm and minimal clinical findings, accounted for a large proportion of disagreements. These observations suggest that further refinement of radiographic thresholds may be necessary to enhance consistency in evaluating intermediate grades of instability.

The high rate of intraobserver reliability, with identical repeated assessments in 96 percent of cases, confirms the internal consistency of the classification and its potential for widespread clinical adoption. Once familiar with the criteria, clinicians appear able to apply the system predictably and reproducibly across different clinical environments, including multicentric research settings.

The present analysis should be interpreted strictly as a reliability study, and no conclusions regarding outcome prediction or clinical effectiveness can be drawn from these data.

### 4.2. Limitations of the Study

This study has several limitations. The relatively small number of evaluators (n = 5) may restrict the generalizability of the findings. Although the inclusion of both specialists and residents allowed a realistic assessment of interobserver variability, a larger cohort of evaluators would strengthen external validity. Another important limitation is the small number of Essex Lopresti injuries (n = 5). Despite the substantial agreement observed in this subgroup, the limited sample size reduces statistical power and restricts broader applicability, and larger prospective cohorts are necessary to determine the performance of the classification in this challenging injury pattern.

The retrospective design introduces additional limitations, including potential selection bias and variability inherent to clinical documentation. Although several clinical and functional parameters were recorded to provide descriptive context, correlations between DRUJ classification grades and functional measures in this cohort require prospective confirmation to minimize confounding influences. As in most reliability studies, prior calibration of observers may have contributed to higher agreement and may not fully reflect reproducibility in settings without structured training.

Existing classification systems for distal radius fractures such as AO, Frykman, and Fernandez have demonstrated inconsistent reproducibility and limited interobserver agreement, partly due to the absence of clearly defined criteria for DRUJ instability [34,35]. Although the present classification integrates clinically relevant imaging and functional criteria, further multicentric validation is required to confirm its broad applicability.

### 4.3. Future Directions

A broader validation of the proposed classification will require prospective multicentric studies that include a larger number of observers and a more diverse spectrum of clinical cases. Such an approach would allow evaluation of the classification across different institutional settings and support its integration into routine clinical practice [34,35,36].

An essential next step is to correlate intraoperative DRUJ instability grades with long term clinical outcomes at 6, 12, and 24 months after surgery. These data would provide evidence regarding the true prognostic value of the classification and support its use as a risk stratification tool. The DRUJ score could thus function as a clinically relevant marker for predicting postoperative complications, persistent instability, or the need for reintervention [37,38].

Developing structured training programs for junior surgeons represents another important direction for improving consistency in the application of the classification. Training modules assisted by artificial intelligence may facilitate recognition of subtle clinical and radiographic patterns associated with each instability grade [39,40].

Advanced imaging techniques such as MRI for TFCC lesions and CT for subtle subluxations may improve diagnostic accuracy in borderline cases [41]. Incorporating these methods into the evaluation protocol may reduce interobserver discrepancies and enhance overall reliability [29].

The classification may be adapted to assess DRUJ instability in other pathological contexts such as degenerative disease, inflammatory arthritis, or postoperative instability, including cases following radial head arthroplasty [1,11,42,43]. Validation in these scenarios would demonstrate the robustness and versatility of the system.

The classification can also serve as a reference standard for future biomechanical research aimed at improving fixation techniques for distal radius fractures or reconstructive procedures for TFCC injuries. Integrating DRUJ scores into cadaveric or experimental studies would allow more objective evaluation of mechanical performance across different fixation methods or implants [44,45].

Integration of this classification into clinical practice would promote standardization of therapeutic decision making and enhance comparability of outcomes across medical centers. Such efforts support the transition to evidence based, personalized medicine, where treatment is tailored to the actual degree of instability and functional prognosis reflected by the DRUJ score [3,46,47,48].

Finally, the recently developed extension of the classification that differentiates between acute and chronic DRUJ instability represents a natural evolution of the system. This component was not included in the present reproducibility analysis and will require independent inter and intraobserver validation. Evaluating this extension in a dedicated methodological study will allow clarification of its potential clinical value and determine whether it should be integrated into the standard form of the classification.

## 5. Conclusions

The proposed classification for distal radioulnar joint instability demonstrated excellent interobserver reliability, with a global Fleiss Kappa value of 0.87, and high intraobserver reproducibility, with identical reassessments in 96 percent of cases. These results confirm that the system can be applied consistently by clinicians with different levels of experience and that its criteria are interpreted uniformly across repeated evaluations.

The findings support the classification as a reliable tool for standardized assessment of DRUJ instability in clinical and research settings. Further prospective multicentric studies are required to evaluate its prognostic value, refine radiographic thresholds in borderline cases, and determine its role in guiding treatment strategies and long-term outcome assessment.

## Data Availability

The original contributions presented in this study are included in the article. Further inquiries can be directed to the corresponding authors.
